# Effects of dietary tryptophan and stocking density on the performance, meat quality, and metabolic status of broilers

**DOI:** 10.1186/2049-1891-5-44

**Published:** 2014-09-26

**Authors:** Bo Wang, Zhizhi Min, Jianmin Yuan, Bingkun Zhang, Yuming Guo

**Affiliations:** 1 State Key Laboratory of Animal Nutrition, College of Animal Science and Technology, China Agricultural University, Beijing 100193, China

**Keywords:** Broiler, Meat quality, Performance, Stocking density, Tryptophan

## Abstract

**Background:**

Highly automated cage-rearing systems are becoming increasingly popular in China. However, a high stocking density can cause oxidative stress and decrease broiler performance. The tryptophan (TRP) derivative 5-hydroxytryptophan (5-HT) has been shown to preserve membrane fluidity in birds suffering from oxidative stress. Therefore, this experiment was conducted to determine the effects of dietary TRP supplementation on performance, breast meat quality and oxidative stress in broilers reared in cages with a high or low stocking density.

**Methods:**

Female Arbor Acres broilers (25-d-old, n = 144) were randomly allocated to 1 of 4 treatments. The birds were fed a diet based on corn, soybean meal, cottonseed meal and corn gluten meal containing either 0.18 or 0.27% TRP and were housed with stocking densities of 11 or 15.4 birds/m^2^ in a 2 × 2 factorial experiment. Broiler performance was evaluated from d 25 to 42. Eight birds from each treatment were slaughtered on d 42 and plasma and breast muscle samples were collected to measure biochemical indices.

**Results:**

A higher stocking density tended to be associated with reduced weight gain (*P* < 0.10), and significantly increased plasma glutamic-pyruvic transaminase (GPT) activity (*P* < 0.001). Increased dietary TRP significantly reduced the activities of lactic dehydrogenase and GPT while increasing total cholesterol in the plasma (*P* < 0.01), reducing drip loss of breast muscle (*P* < 0.10) and improving feed efficiency (*P* < 0.10).

**Conclusions:**

An increase in dietary TRP, 1.5-fold higher than the standard supplementation level, can alleviate oxidative stress as well as improve welfare and feed efficiency in broilers reared in cages with a high stocking density.

## Background

Increased attention is being paid to animal welfare and meat quality, factors that are strongly affected by stocking density [1]. A low stocking density is thought to be associated with high product quality, although it produces less meat and can therefore result in economic losses [1]. In contrast, a high stocking density can increase the quantity of broiler meat produced per unit area [2]. However, previous studies have shown that high stocking densities affect the amount of time broilers spend walking [3], decrease locomotor activity [4,5], as well as increase the temperature, moisture, and ammonia concentrations in the chicken house, and cause leg and hock problems [6-8]. In addition, a high stocking density can increase disturbances [5] and conflicts between birds [9], and lead to apparent fearfulness [8,10]. Other problems associated with a high stocking density include increased heterophil:lymphocyte ratios [11], and increased plasma concentrations of glutathione peroxidase, leading to oxidative stress [4,8,12]. Thus, high stocking densities result in reduced growth, impaired feed efficiency, and poor quality of life for broilers [4,13].

Tryptophan (TRP), the rate-limiting substrate of 5-hydroxytryptamine (5-HT), is synthesized in the brain and competes with large neutral amino acids (LNAA) to cross the blood–brain barrier [14]. TRP supplementation significantly increased 5-HT turnover in the hippocampus and archistriatum and tended to do the same in the remainder of the forebrain [14]. Previous studies have shown that birds fed low-TRP diets exhibited abnormal behavior and signs of stress [15]. An increase in dietary TRP has been shown to reduce aggression, and alleviate stress in livestock and poultry [16,17], reduce feather-pecking behavior and reduce hysteria in layers [18].

Excessive TRP reduced weight gain in weaned piglets [19] and rats [20]. However, no studies have investigated the effects of stocking density in relation to different concentrations of dietary TRP especially in broilers raised using cage rearing systems. Multi-storey cages or highly automated cage-rearing systems are becoming increasingly popular in China and other countries because they enable convenient management and removal of excreta and minimize bird contact with wastes, which reduces ammonia concentrations and improves chicken welfare. However, whether TRP supplementation can reduce stress and improve the welfare of broilers and quality of meat produced under high stocking densities is unknown. In this study, the effect of TRP supplementation on performance, breast meat quality, and the oxidative stress status of broilers reared in cages under high stocking density was investigated.

## Materials and methods

### Bird management

This protocol used in this study was approved and conducted in accordance with the guidelines of the Animal Ethics Committee of China Agricultural University (Beijing, China). Day-old female broiler chicks (Arbor Acres Plus), obtained from a commercial hatchery (Aviagen Group, Beijing, China), were raised in the middle floor of a 3 tier set of overlap cages. Individual cages were 70 cm long × 65 cm wide × 38 cm tall, and contained two nipple drinkers. A galvanized iron trough, hanging outside of the cage, was used to feed the broilers. Excreta was collected in trays under each cage, and was removed every 2 or 3 d to decrease ammonia concentrations in the broiler house.

Broilers were raised according to the Arbor Acres Broiler Management Guide (Aviagen Group, Beijing, China). The temperature was maintained at 34°C on day 1, and was gradually reduced to 22°C by day 21. Feed was offered ad libitum and water was freely available at all times. The relative humidity was maintained at 40–65%. The experiment was performed during winter and open windows were used to achieve cross-ventilation.

### Experimental design and diets

The trial used a 2 × 2 factorial arrangement, involving 2 stocking densities and 2 concentrations of TRP. The normal stocking density was 5 birds/cage (11 birds/m^2^), and the high stocking density was 7 birds/cage (15.4 birds/m^2^), according to Sørensen et al. [21]. The normal TRP concentration was defined as 0.18%, on the basis of NRC (1994) nutritional requirements for broilers [22], and the high TRP concentration was 0.27% (1.5-fold higher than the normal concentration). Since the effects of stocking density occur primarily during the growing period [6], the experiment commenced when the broilers were 25 days of age. From days 1 to 24, the birds were fed a commercially prepared mash diet. On day 25, the birds were individually weighed, and 144 birds with similar body weights, were randomly assigned to 24 cages. The difference in the average body weight among the cages was <25 g. From days 25 to 42, birds were fed the experimental diets in mash form (Table 1).

**Table 1:** **Composition and nutrient level of diets** (**air**-**dry basis**)

**Item**	**0.18% TRP**	**0.27% TRP**
Ingredients, %		
Corn	64.64	64.54
Soybean meal	16.12	16.12
Cottonseed meal	6.00	6.00
Corn gluten meal	6.00	6.00
Soybean oil	3.23	3.23
Limestone	1.15	1.15
Dicalcium phosphate	1.56	1.56
NaCl	0.35	0.35
Vitamin premix^1^	0.02	0.02
Trace mineral premix^2^	0.20	0.20
*DL*-Met (98%)	0.04	0.04
*L*-Lys · HCl (78%)	0.40	0.40
*L*-Thr	0.06	0.06
Choline chloride (50%)	0.16	0.16
*L*-Trp (98%)	0.01	0.10
Nutrient levels	0.06	0.06
AME, Mcal/kg		
Crude protein,%	3.10	3.10
Calcium, %	19.00	19.00
Available phosphorus, %	0.88	0.88
Nutrient levels	0.40	0.40
Amino acid content		
Lys, %	1.00	1.00
Met, %	0.38	0.38
Thr, %	0.64	0.64
Trp, %	0.18	0.27

^1^The vitamin premix provided the following per kilogram of diet: vitamin A, 10,000 IU; vitamin D_3_, 2,400 IU; vitamin E, 20 IU; vitamin K_3_, 2.00 mg; thiamin, 2.00 mg; riboflavin, 6.40 mg; pyridoxin, 3.00 mg; VB_12_, 0.02 mg; folic acid, 1.00 mg; pantothenic acid, 10.00 mg; nicotinic acid, 30.00 mg; biotin, 0.10 mg.

^2^The mineral premix provided the following per kg of diet: Cu, 8 mg; Fe, 80 mg; Zn, 60 mg; Mn, 100 mg; I, 0.35 mg.

### Measurements

#### Broiler performance

From days 25 to 42, mortality, culling and feed intake were recorded daily for each pen. On day 42, BW was recorded on a per cage basis to calculate the final BW and BW gain. Daily feed intake and feed efficiency were corrected for mortality.

#### Metabolic status

On day 42, blood samples were collected into heparinized tubes from the wing vein of 8 birds per treatment. The samples were centrifuged for 10 min at 3,000 × *g*, and plasma was collected and frozen at -40°C for subsequent analysis of lactic dehydrogenase (LDH), glutamic-pyruvic transaminase (GPT), glutamic-oxalacetic transaminase (GOT), glucose (GLU), triglyceride (TG), and total cholesterol (TC). The analyses were performed using commercial analytical kits according to the manufacturer’s recommendations (Jian Cheng Bioengineering Institute, Nanjing, China).

#### Breast meat quality

On day 42, 8 birds closest to the average BW for each treatment were slaughtered. The left breast muscle was dissected, and breast muscle yield (without bone or skin) was measured as the ratio of 2 times the Pectoralis major and Pectoralis minor muscle mass to the final BW of the bird. The pH_45_ min post-mortem (pH_45_) was measured using a TESTO 205 pH meter (Testo Limited, Alton, Hampshire, UK) by inserting the electrode into three different points of the right section of the Pectoralis muscle and the average values were used for statistical analysis. Crude fat was measured by ether extraction of a 20-g sample of breast meat. An additional 20 g sample was placed in a polyethylene bag and drip loss was determined after 2 d of storage at 4°C [23].

### Statistical analysis

The data was analyzed to assess the interactive effects of stocking density and TRP concentrations using the General Linear Model (GLM) of SPSS 15.0 [24]. Percentage data was converted to degrees before Analysis of Variance was conducted and was subsequently back-transformed to generate an estimate of the standard error of the mean (SEM). Significance was set at *P* < 0.05, and trends were recognized at *P* < 0.10.

## Results

### Performance

Only one bird died during the experiment and as a result, mortality is not shown in the tables. There were no significant differences in the initial bodyweight among the experimental groups. However, increased stocking density tended to reduce the final BW and bodyweight gain (*P* < 0.10) (Table 2). There were no significant differences in feed efficiency or feed intake. The addition of dietary TRP tended to decrease feed efficiency (*P* < 0.10) but had no effect on BW gain or feed intake. There were no significant interactions between dietary TRP concentrations and stocking density on the final BW, BW gain, or feed intake. However, high stocking density in combination with increased TRP supplementation significantly lowered feed efficiency compared with a high stocking density with normal TRP supplementation (*P* < 0.05), while a lower stocking density in combination with increased TRP supplementation did not significantly affect feed efficiency.

**Table 2:** **Effects of stocking density and tryptophan** (**TRP**) **level on broiler performance**^**1**^

**Stocking density, birds/m**^**2**^	**Tryptophan Level, %**	**Initial Bodyweight, g**	**Final Bodyweight, g**	**Daily bodyweight Gain, g**	**Feed Intake, g/d**	**Feed efficiency**
**Stocking density × tryptophan level**						
11.0	0.18	778	1,960	69.53	127.2	1.83^b^
11.0	0.27	780	1,961	69.47	126.5	1.82^b^
15.4	0.18	781	1,887	65.00	123.5	1.90^a^
15.4	0.27	781	1,939	68.06	123.2	1.81^b^
SEM		5.6	76.2	4.250	6.30	0.059
**Stocking density**						
11.0		779	1,963	69.65	126.8	1.82
15.4		781	1,913	66.59	123.4	1.85
SEM		1.1	16.4	0.900	1.28	0.012
**Tryptophan level**						
0.18%		780	1,924	67.24	125.3	1.86
0.27%		782	1,950	68.76	124.9	1.82
SEM		1.1	15.5	0.057	1.28	0.015
*P*-values						
Stocking density		0.351	0.084	0.089	0.214	0.144
TRP level		0.891	0.564	0.509	0.851	0.052
Density × TRP		0.659	0.265	0.375	0.938	0.045

^1^Each mean represents 6 replicates for each treatment with each replicate containing 5 (11.0 birds/m^2^) or 7 birds (15.4 birds/m^2^).

^a,b^Means in the same row with different superscripts are significantly different (*P* < 0.05).

### Breast meat quality

Increased stocking density had no significant effect on breast muscle yield, fat content, pH_45_, or drip loss (Table 3). Increased TRP concentration tended to reduce drip loss in the breast muscle of broilers (*P* < 0.10). There was no influence of TRP concentration on breast muscle yield, breast fat content, or pH_45_. Interactions between TRP concentration and stocking density were not significant for fat content, pH_45_, or drip loss. However, the high stocking density in combination with the low level of TRP supplementation tended to produce significantly higher breast muscle yield compared with the other treatments.

**Table 3:** **Effects of stocking density and tryptophan level on breast meat quality of broilers**^**1**^

**Stocking density, birds/m**^**2**^	**Tryptophan level, %**	**Body weight, kg**	**Breast muscle**			
			**Yield, %**	**Fat content, %**	**pH**_**45**_^**2**^	**Drip loss, %**
**Stocking density × tryptophan level**						
11.0	0.18	1.87	14.25	0.79	6.07	9.17
11.0	0.27	1.98	14.82	0.69	6.11	8.50
15.4	0.18	1.87	15.45	0.66	6.16	11.60
15.4	0.27	1.82	14.43	0.67	6.13	8.16
SEM		0.228	1.083	0.121	0.195	2.502
**Stocking density**						
11.0		1.93	14.54	0.74	6.09	8.84
15.4		1.85	14.94	0.66	6.14	9.63
SEM		0.046	0.173	0.025	0.040	0.511
**Tryptophan level**						
0.18%		1.87	14.85	0.73	6.11	10.14
0.27%		1.90	14.62	0.68	6.12	8.33
SEM		0.046	0.173	0.025	0.040	0.511
***P *** **Value**						
Stocking density		0.405	0.356	0.130	0.533	0.425
TRP level		0.745	0.599	0.351	0.907	0.077
Density × TRP		0.405	0.077	0.302	0.710	0.258

^1^Each mean represents 8 birds for each treatment.

^2^Note: pH_45_ represents pH value for breast meat 45 min after slaughter.

### Metabolic status

A high stocking density tended to increase plasma GLU (*P* < 0.10), and significantly increased plasma GPT activity (*P* < 0.001). There were no significant differences in the activity of LDH and GOT or in the plasma levels of TG and TC.

Increased dietary TRP significantly increased plasma TC (*P* < 0.05), and decreased LDH and GPT activities (*P* < 0.01). There were no significant differences in GOT activity or in GLU and TG levels between the TRP treatments. Significant interactive effects were observed between TRP concentration and stocking density for plasma GLU (*P* < 0.01), LDH activity (*P* < 0.05), and GPT activity (*P* < 0.001). A high stocking density in combination with increased dietary TRP significantly increased GLU content compared with the other treatments and significantly decreased GPT activity compared with the normal TRP treatment with a high stocking density, although the level did not differ significantly from the low stocking density treatments. A high stocking density in combination with increased TRP supplementation significantly decreased LDH activity compared with the low-TRP treatment, but showed no significant differences from the low stocking density treatment.

## Discussion

In this study, increased stocking density tended to reduce weight gain of broilers (Table 2), a finding that is consistent with previous studies that demonstrated that a high stocking density is associated with decreased BW [4], especially during the growing period [7]. However, Estevez [25] suggested that the recommended stocking density could be increased to approximately 18 birds/m^2^ without any major adverse effects on the final BW. Ekstrand and Carpenter [26] proposed that the response to changes in stocking density depended on environmental variables. Differences in the environment provided to chickens by poultry producers might have greater effects on welfare than stocking density [27].

Clements [28] reviewed numerous articles on stocking density and animal welfare and concluded that there was no significant relationship between stocking density and behavior, leg problems, or skin damage. Under deep litter conditions, growth rate was significantly reduced when stocking density exceeded approximately 30 kg/m^2^ live weight [7]. However, the effects of litter and stocking density on growth were attenuated when ventilation was increased or when broilers were raised with perforated flooring.

Increased litter temperature has been identified as a causal factor for reduced growth rate [29]. High stocking densities might contribute to reduced broiler performance because of high temperatures and reduced airflow at the bird level [26]. The reduction in BW gain at a stocking density of 15.4 birds/m^2^ compared with that at a stocking density of 11 birds/m^2^ might be related to the cage environment (e.g., air flow, temperature, and cage area). Cage area might affect broiler performance, which also affects locomotor activity [4,5]. Suitable stocking density when birds are raised in cages might differ from the stocking density required when birds are raised with open litter floor or perforated flooring. The suitable stocking density of broilers raised in 3-tier-overlap cages would be <15.4 birds/m^2^ if simple cross ventilation were used by opening windows during the winter.

Increasing the stocking density of female birds from 11 to 15.6 birds/m^2^ did not affect breast meat yield (Table 3), which is in agreement with the findings of Moreira et al. [30] who showed that densities between 10 and 16 birds/m^2^ did not affect broiler meat quality characteristics (cooking loss, shear force, and pH) in Ross 308, Cobb 500, and Hybro PG commercial strains. Thomas et al. [10] also showed that stocking densities of 10, 15, and 20 birds/m^2^ had no influence on carcass characteristics. However, Rilgili and Hess [31] found that increased stocking density could significantly reduce carcass quality and breast meat yield in fast-growing male birds, whereas slow-growing female birds were not affected. Simitzis et al. [4] showed that high stocking density decreased intramuscular breast fat.

The effects of stocking density on meat quality might be related to growth rate. Puron et al. [2], showed that similar masses of meat were produced when housing density exceeded 17 male or 19 female birds/m^2^ which was close to the densities recommended to minimize the adverse effects of crowding on broiler welfare. The stocking densities of female broilers in the current study (11 and 15.4 birds/m^2^) were lower than those used by Puron et al. [2], and slow growing female broilers were used in the current study, similar to the experiment of Rilgili and Hess [31] which might explain the lack of significant differences in breast meat quality between the 2 stocking densities.

Simsek et al. [12] compared stocking densities of 6 and 13 birds/m^2^ and found that a high stocking density decreased feeding time and caused oxidative stress. Simitzis et al. [4] showed that a high stocking density was associated with decreased locomotor activity and increased indicators of physiological (heterophil:lymphocyte ratio and bursa weight), and oxidative stress. However, other researchers have shown that stocking density did not affect the major physiological stress indicators, including plasma glucose and cholesterol [32] and fecal corticosterone concentrations [8,27]. In this study, a high stocking density did not influence the levels of TG or TC or the activities of LDH and GOT in the plasma, which is in agreement with previous studies [7,33]. However, an increased stocking density significantly increased GPT activity in the plasma.

Serum GPT and GOT are commonly measured clinically as a part of diagnostic liver-function tests to determine liver health. Levels of serum GPT and GOT generally increase with muscle or liver damage [34]. The significantly increased GPT activity indicates that a high stocking density might cause oxidative lesions, which is in agreement with the findings of Simsek et al. [14]. However, whether or not increased stocking density causes oxidative stress with no change in LDH activity related to the stress level needs further research.

Previous studies have shown that the TRP derivative 5-hydroxytryptophan could help preserve membrane fluidity in animals experiencing oxidative stress [35]. Another important TRP derivative, melatonin, was found to have anti-oxidative properties [36,37]. In the current study, increased dietary TRP significantly decreased the TC and the activities of LDH and GPT in plasma (Table 4). Increased plasma GPT activity under a high stocking density was moderated by the increase in dietary TRP concentration, suggesting that increased dietary TRP could help prevent liver injury resulting from a high stocking density.

**Table 4:** **Effects of stocking density and tryptophan** (**TRP**) **level on plasma parameters of broilers**^**1**^

**Stocking density, birds/m**^**2**^	**Tryptophan Concentration, %**	**LDH**^**2**^**, U/L**	**GLU, mg/dL**	**TG, mg/dL**	**TC, mg/dL**	**GPT, U/L**	**GOT, U/L**
**Stocking density × tryptophan level**							
11.0	0.18	24,219^ab^	239.1^b^	39.49	106.1	2.88^b^	32.92
11.0	0.27	22,317^b^	228.2^b^	51.48	121.2	3.83^b^	31.95
15.4	0.18	28,028^a^	232.6^b^	48.30	104.8	9.10^a^	32.60
15.4	0.27	19,695^b^	256.9^a^	58.97	121.2	5.05^b^	32.33
SEM		5,106.0	16.66	17.956	16.67	2.595	4.575
**Stocking density**							
11.0		23,268	233.6	45.49	113.6	3.36	32.43
15.4		23,861	244.8	53.63	113.0	7.08	32.47
SEM		1,042.3	3.40	3.665	3.40	0.529	0.934
**TRP level**							
0.18%		26,123	235.9	43.89	105.4	5.99	32.76
0.27%		21,006	242.5	55.23	121.2	4.44	32.14
SEM		1,042.3	3.40	3.665	3.40	0.529	0.934
***P *** **value**							
Stocking density		0.742	0.053	0.272	0.925	<0.001	0.987
TRP level		0.009	0.232	0.132	0.023	0.001	0.761
Density × TRP		0.024	0.004	0.928	0.925	<0.001	0.863

^1^Each mean represents 8 birds for each treatment.

^2^*LDH*: lactic dehydrogenase; *GPT*: glutamic-pyruvic transaminase; *GOT*: glutamic-oxalacetic transaminase; *GLU*: glucose; *TG*: triglyceride; *TC*: total cholesterol.

^a,b^Means in the same row with different superscripts are significantly different (*P* < 0.05).

The cytoplasmic enzyme LDH is widely used as a marker of organ or tissue lesions in toxicology and clinical chemistry. LDH is generally associated with cellular metabolic activity, which is inhibited or elevated under oxidative stress [38]. The significant decrease in LDH activity observed in the present study with dietary supplementation of 0.27% of TRP under the high stocking density conditions, indicates that TRP level can reduce the stress caused by a high stocking density.

Corzo et al. [15], showed that supplemental TRP increased plasma glucose in a linear manner, whereas other physiological stress variables were unaffected by dietary TRP. Plasma glucose exhibited a linear response to TRP supplementation, most likely due to adrenergic-driven glycolytic factors, unlike the gluconeogenic events associated with physiological stress [34].

Previous study showed that a high stocking density increased plasma concentrations of glutathione peroxidase, leading to oxidative stress [4,8,12]. TRP treatment significantly increased the TRP/LNAA ratio in the plasma of the chicks. Furthermore, TRP treatment increased baseline and stress-induced levels of plasma corticosterone, which inhibits glycolysis [16]. Supplementation with the high concentration of TRP significantly increased TC levels compared with low TRP concentrations (Table 4), which also indicates that increased TRP could help alleviate oxidative stress. This might explain why high concentrations of TRP tended to alter feed efficiency. This indicates that increased TRP concentration could help to improve feed efficiency under high stocking densities.

Drip loss can indicate quality deviations associated with the rate and extent of postmortem glycolysis in muscle tissue. The volume of drip loss is related to the lipid peroxide content in muscle [39]. Our finding that high TRP supplementation reduced drip loss under a high stocking density indicates that administration of TRP could lead to a reduction of lipid peroxide and glycolysis values caused by stress due to a high stocking density, thus improving meat quality.

In conclusion, increasing the stocking density from 11 to 15.4 birds/m^2^ caused oxidative stress in broilers and tended to reduce their performance. Supplementation with TRP at concentrations 1.5-fold higher than the standard dose commonly supplemented could alleviative oxidative stress caused by stocking density and could improve broiler welfare, meat quality, and feed efficiency.

## Abbreviations

BW: Body weight; GLU: Glucose; GOT: Glutamic-oxalacetic transaminase; GPT: Glutamic-pyruvic transaminase; LDH: Lactic dehydrogenase; LNAA: Large neutral amino acids; TC: Total cholesterol content; TG: Triglyceride; TRP: Tryptophan.

## Competing interests

The authors declare that they have no competing interests.

## Authors’ contributions

BW and ZM carried out the experiments, JY wrote the manuscript, BZ, and YG participated in the design of the study. All authors read and approved the final manuscript.
